# Nurse managers implementing the lean management system: A qualitative study in Western Canada

**DOI:** 10.1111/jonm.12898

**Published:** 2020-02-25

**Authors:** Sonia A. Udod, Judy Boychuk Duchscher, Donna Goodridge, Thomas Rotter, Petrina McGrath, Anna Dawn Hewitt

**Affiliations:** ^1^ Rady Faculty of Health Sciences College of Nursing University of Manitoba Winnipeg MB Canada; ^2^ School of Nursing Thompson Rivers University Kamloops BC Canada; ^3^ College of Medicine Saskatoon SK Canada; ^4^ Health Quality Programs Queen's University Kingston ON Canada; ^5^ Quality and Safety Saskatchewan Health Authority Saskatoon City Hospital Saskatoon SK Canada; ^6^ Saskatchewan Health Authority Tisdale SK Canada

**Keywords:** health care delivery, health care systems, leadership, nurse administrators, nursing supervisory

## Abstract

**Aim:**

This study explores the perceptions and experiences of nurse managers involved in implementing the Lean management system in a Western Canadian province.

**Background:**

The provincial government of Saskatchewan, Canada, implemented a multimillion‐dollar investment in the Lean management system to transform health care delivery by reducing waste and increasing efficiency of processes and outcomes.

**Methods:**

This qualitative exploratory study employed semi‐structured interviews with 14 nurse managers in urban and rural health regions in one Canadian province.

**Results:**

Six themes outline the difficulties nurse managers experienced in juggling role responsibilities alongside a poorly implemented change system with scarce resources.

**Conclusion:**

The results showed tensions in the implementation of a Lean model adapted in the context of health care organisations. The expectations for nurse managers to be pivotal players in the implementation of transformative health care practices that promote and sustain strategies to reduce waste, improve coordination and increase patient safety require investment in leadership development.

**Implications for Nursing Management:**

Lean management systems significantly impact the roles of nurse managers who require adequate resources and training to successfully adapt. The results of this study may be used for more effective support mechanisms for nurse managers.

## 1 BACKGROUND

Nurse managers (NMs) play an integral role in building and sustaining workplaces conducive to improved productivity, cost, quality and timely delivery of health care services (Johnson, Smith, & Mastro, 2012; Titzer & Shirey, 2013). Within this context, a host of health care trends co‐exist in Canada, such as rapidly intensifying costs, inefficiencies, lack of consumer‐centeredness and concerns about the overall quality and safety of health care (Fine, Golden, Hannam, & Morra, 2009; Steed, 2012). These constraints have challenged the capacity of NMs to perform their role and meet organisational expectations. The Toyota Production System, also known as the Lean management system, has been identified as a viable and sustainable solution for the growing cost, quality and efficiency challenges faced by health care leaders (Fine et al., 2009; Graban, 2009; Knowles & Barnes, 2013).

Lean is a complex set of philosophies, assessment and improvement activities aimed at maximizing value by reducing waste and effort, and increasing production flow (Poksinska, Swartling, & Drotz, 2013). When transferred to health care, Lean is described as comprising two defining characteristics: (a) *philosophies*, or a set of principles, aimed at transforming the workplace culture and focusing on continuous improvement by eliminating waste, improving flow of patients, providers and suppliers, and processes; and (b) *assessment activities* or analytic tools that identify waste and improvement activities (Rotter et al., 2014). Ultimately, the aim of Lean is to facilitate access to cost‐effective, high‐quality and innovative health care that puts the patients and direct‐care providers at the centre of determining what care is optimal, and what essential resources are required to achieve the best patient outcomes (Kinsman et al., 2014). However, Hines, Found, Griffiths, and Harrison (2008) suggested the intent, though enticing, was reported by many institutions to be unsustainable. Critics have argued that the industry origins of Lean make its principles less transferable to the dynamic environment of contemporary health care, because patient needs are not analogous to production flow (Dickson, Singh, Chueng, Wyatt, & Nugent, 2008; Drotz & Poksinska, 2014).

Numerous studies report that leadership is the fundamental ingredient for an effective Lean health system transformation (Aji, Visse, & Widdershoven, 2015; Steed, 2012). Lean leadership is a set of leadership practices, tools and behaviours that focus on processes to drive improvements (Mann, 2009). Mann (2009) states that failing to change leadership practices may be the reason Lean initiatives fail and why organisations are unable to improve value‐producing processes. For NMs, Lean emphasizes the importance of their presence on the unit for the purpose of being visible, coaching and reinforcing new practices to reinforce behaviours and sustain change (Aji & Rapsaniotis, 2017; Mann, 2009). The NM is instrumental in defining and updating outcome metrics, focusing on root cause analysis and problem solving, and conducting value stream mapping (Goodridge, Westhorp, Rotter, Dobson, & Bath, 2015; Mann, 2009). Even though front‐line leadership is considered key to successful Lean implementation, little research exists that has focused on the role of NMs in Lean implementation. Therefore, in this study we aimed to explore the experiences and perceptions of NMs to better understand the implementation of the Lean system and its impact on nursing leadership roles.

## 2 METHOD

### 2.1 Setting

This study was conducted within a publically funded health care system in the province of Saskatchewan, Canada (population 1 million), after government implementation of a multimillion‐dollar investment in the Lean management system (Kinsman et al., 2014). All 13 health regions in the province, comprised of secondary and tertiary hospitals, community health centres, ambulance and emergency services, support care, home care, mental and rehabilitation, were included in the roll‐out.

In 2012, the province decided to use a specifically branded approach to Lean, engaging John Black and Associates to assist with implementation across the entire province (; Rotter et al., 2014). The initial phase of Lean implementation focused on senior leadership training and the creation of Kaizen Promotion Offices (KPOs) that provided supportive infrastructure to coordinate and advance the transformation. By 2012, phase 1 regions commenced Lean implementation in hospitals, followed by phase 2 implementation (2014) in primary care in rural and remote regions (Rotter et al., 2014).

The initial phase of the process improvement initiative promoted the implementation of Releasing Time to Care™ (RTC). Lean principles were used with RTC to facilitate the engagement of front‐line nurses in improving quality of care and reducing waste in acute hospitals (Hamilton et al., 2014). This project was followed by the establishment of Lean KPOs in five health regions. The KPOs aimed to train and certify Lean leaders, including NMs, to spearhead quality improvement work. The Lean management system was introduced into the health care environment in combination with a strategic management and policy deployment system, called Hoshin Kanri, and daily visual management (e.g. daily huddles and visibility walls) (Rotter et al., 2014).

The study received approval from the ethics boards of the University of Saskatchewan and two health regions.

### 2.2 Study design and participants

A qualitative exploratory approach was used to better understand the experiences of NMs and their perceptions of factors that facilitated or impeded the sustainability of the Lean management system in acute care settings. Telephone contact was made with health region senior nurse administrators to obtain contact information for NMs who met the eligibility criteria. A letter of invitation via email was sent to senior nurse leaders for distribution to eligible NMs in the health region, inviting them to participate in the study. Inclusion criteria included the following: (a) must be a registered nurse; (b) have knowledge and participation in Lean training; (c) work in an urban or rural health care facility in the province of Saskatchewan; and (d) possess minimum one‐year experience as a NM. Managers who met the eligibility criteria were contacted to participate in an individual interview. This purposive sampling (Patton, 2015) strategy allowed for the recruitment of participants with a diversity of organisational and individual experience. Prior to each interview, participants provided informed, written consent.

Data were collected from 14 NMs employed in urban and rural health regions in the province. Participants were employed in eight different acute care facilities within two health care regions. The participants were almost exclusively female (94%) and ranged in age from the 31 to the 61+ years of age. Almost two‐thirds of participants supervised more than 90 employees (see Table 1).

**Table 1:** Characteristics of nurse managers (*n* = 14)

Gender	
Female	13
Male	1
Age (years)	
21–30	
31–40	4
41–50	3
51–60	7
61–69	
Education	
Diploma	1
Bachelor degree	13
Graduate degree	6
Nursing experience	
<5 years	1
5–10 years	0
11–15 years	2
16–20 years	1
21–35 years	10
Time in current manager role	
<5 years	11
5–10 years	2
11–20 years	1
20–25 years	
More than 25 years	
Number of employees supervised	
<20 persons	1
21–50	1
51–100	6
101–200	3
More than 200	3
Health setting	
Urban	8
Rural	6

### 2.3 Data collection and analyses

A semi‐structured interview schedule was developed based on behaviours and managerial practices of NMs that facilitate or hinder Lean implementation. Individual interviews were conducted by SU, JBD and the research assistant in the participant's office and/or by telephone with each interview lasting from 40 to 60 min, then audio‐recorded and transcribed verbatim. Data analysis was based on Braun and Clarke's (2006) framework for thematic analysis, which involved the search for, and identification of, common threads across all the data. Operational definitions were written for all codes in a codebook. Analytic processes from grounded theory such as concurrent data generation and analysis and constant comparison of data were used. Then, all themes were reviewed and emerging themes were examined in relation to the codes and the entire data set. The team then refined the specific details of each theme, generating clear definitions and themes. Analysis continued until categories provided a comprehensive understanding of the phenomenon, and further data became redundant. The final phase of analysis focused on theme refinement and identification of NM perceptions of factors that either facilitated or impeded the Lean management system transformation.

Trustworthiness was used to support the rigour of the study in the following ways (Lincoln & Guba, 1985). Credibility was met through member checks where participants had the opportunity to provide additional comments in the interview to strengthen the accuracy of their interpretations. Dependability was met through an audit trail, and confirmability was met through investigator triangulation by independent coding and analysis of the first three interviews by two researchers and the research assistant through an iterative feedback process until consensus was reached. Transferability was met through thick rich word description to demonstrate the quality of the data.

## 3 RESULTS

We identified six themes related to NMs' experiences and perceptions of their role during the Lean implementation in their health units.

### 3.1 Absence or limited Lean training and preparation

Participants experienced varying levels of training, ranging from intense week‐long sessions to self‐study programmes. A lack of uniform educational training made it difficult to integrate or put Lean knowledge into action for implementing change. For example, some participants toured Lean managed hospitals in the United States; however, as finances grew more constrained, other participants reported their training consisted of attendance at a local one‐day workshop, then training was reduced to a half‐day workshop and finally reduced to a self‐study programme. In other words, educational opportunities to learn the Lean philosophy and principles, and tools for practice were scaled‐back as the change initiative gained momentum and financial pressures occurred. A participant explained:

It was pretty intense, overwhelming and very intimidating but it was taught by John Black and Associates and it was very regimented and very strict guidelines … so we were in a warehouse like every day for education, you know, lectures, we were given modules in these actual books… (#11)

Like many participants, another NM expressed her lack of understanding of Lean after attending a scaled‐back version of training: ‘So I did that basic session, it was an afternoon…I mean it's, too general to really be of any value… [training]it was four hours…’ (#9).

Participants reported varying levels of training and the depth in which Lean principles, methods and tools were communicated. As such, it was difficult to create a foundation to initiate and drive forward the improvement work.

### 3.2 Triaging time

Participants perceived that given the complex and pressured nature of the health care environment and a mounting managerial workload, they found it difficult to incorporate Lean as part of their role amid competing priorities. A participant explained:

I don't think we fully understood what that commitment would be…every time I came back to my desk my work remained; the idea being was the first week [time devoted to Lean] was 25% then it was 50%, then 75%, then 100% week…they [senior leaders] didn't really tell you that was gonna carry on. (#12)

Another participant explained how the time pressures on her role resulted in choosing a project that would not require an extensive time commitment:

‘…they require you to do a project on everything and that two week time frame is like, okay I have to get it done. I [need to] randomly pick something that is easy to do, and so you're just going through the motions, it's kind of meaningless and that's frustrating’. (#3)

Participants expressed concern in being afforded the time to understand and integrate Lean as quickly as senior leaders expected. They frequently favoured managerial priorities that required immediate action over engaging with Lean activities that were not well understood. Overall, participants perceived themselves as unable to regularly and meaningfully participate in Lean.

### 3.3 Limited financial resources and limited Lean uptake

The majority of participants described limited or circumscribed financial and human resources associated with poor uptake of Lean. Financial constraints in health regions were exacerbated with the introduction of Lean because staff resources were required to replace leaders attending workshops, or staff members who took part in ‘Kaizen’ events (rapid improvement training events). A participant explained why she worked alone to introduce Lean:

You have to be able to have the resources in smaller communities to pull staff in, we don't have that. We can barely staff our facilities on the best of days so to bring them in to do a 5s [optimizing productivity by maintaining an orderly workplace]… so that's been one of the reasons why I do a lot of my projects alone. (#3)

Another participant talked about how the Lean transition failed to improve patient services:

I just think that of all the money that was spent on Lean, it seems like there's money for that right? …but, if we need money to bring in staff to do an extra bath…. there's no money. (#4)

Participants perceived Lean as a management system largely focused on cost‐cutting. With poor training and support, they were limited in their ability to influence the uptake of Lean.

### 3.4 Ambivalence towards Lean

Participants referred to Lean as additional work, but they did not consistently perceive it to be a meaningful feature of their role. There was a push‐pull perspective conveyed by participants that acknowledged the value of what Lean could theoretically achieve, such as decrease waste and potentially improve care at the bedside. However, the manner in which Lean was implemented in their units and organisations did not support shared decision making with leaders or staff or take into consideration existing workloads. A participant explained how the lack of consultation negatively influenced her buy‐in:

… I did believe and still do believe that this is the way to go [Lean] and …I think there's a positive way to do this, yet I felt very mixed that I wasn't the chosen one from the start and now we need somebody who could commit to it [Lean] on short notice so it didn't provide me an opportunity to say, ‘okay I've got 3 weeks to kind of get myself ready here I can finish this, hand this off, line this up.’ (#12)

For some participants, a lack of trust in how Lean could make a difference to patient care and workflow appeared to undermine the initiative:

[We are] willing to contribute but I think we don't feel like we have the time, energy or skills to be able to deliver the product ourselves without a lot of support. In my mind I feel like what they want to do is be able to train us and we'll be able to do all those operations ourselves and I just do not see that happening… (#1)

While participants expressed the value of Lean in theory, the process of enacting Lean created uncertainty and stress with pre‐existing burdensome workloads. Participants believed senior management would inevitably change their minds, adopting another ‘fad’ as the quality management strategy. This perception created uncertainty and a subtle resistance within daily work, as they weighed the cost of investing time and energy into shifting demands.

### 3.5 Fragmented implementation

Participants described the enactment of Lean as random bursts of activities, sometimes deemed ‘easy to do’, rather than meaningfully building and supporting improvement processes. Moreover, Lean was perceived as lacking incremental or sequential implementation, and seemingly devoid of an overall vision. A participant said, ‘I think what was frustrating [was that] it was a very choppy and confusing implementation’. (#7) For example, teams comprised of leaders and staff from various disciplines and units or departments would use a Lean tool to address a workflow issue. Participants described this activity as ‘checking off a box’ to complete a requirement of the transition. When a change activity was completed, few staff had usually been included in the event, and as a result, typically there was not enough momentum to sustain the change.

Another participant explained the challenge in implementation given her limited training:

I haven't found it [training] really helpful… I've been very honest, that I don't know if it [Lean] works, because I haven't been able to implement it the way you're supposed to. Because of my staffing shortages, because I probably don't buy into it a lot either so you know you're (laughs) doing it but know there's a sense that in ten years we'll be doing something else …and the leaders don't necessarily do it… (#2)

Another participant shared how she was completing her training and had been engaged with Lean for approximately five years. She described how reporting tools were used to improve efficiencies:

We were just talking at our OH&S and they said well use this ‐ And I said well nobody's ever told us that we could use this [tool]. Because the Kaizen promotion people know how to use the tool it just flows from one to the other, but we have to know a little bit about this, how our med incident will flow together. I have to think really hard when I'm going to use these…. Okay now I have to run and do these silly audits, who's going to look at these silly audits that I just did? I don't send them anywhere, I just fold them up and I put them in my desk…

R: You don't need to send them anywhere or share them with anybody?

P: At one time I was sending them to our Kaizen promotion office

R: What did they do with them?

P: I have no idea. (#5)

Limited knowledge about how to use the tools and techniques of Lean created a disjointed process for implementation. Activities focused on micro‐level efficiencies at the unit level to achieve a quick win and inform management the change was completed.

### 3.6 Relationship building

Participants understood their NM role within the context of Lean revolved around being visible on the unit (Gemba walk) and listening to staff ideas about how processes and systems could be improved. But it was not clear from most participants how their interactions on the unit helped resolve a problem or improved a process. A participant described a core tool: ‘…we have these 5‐10 minutes huddles every morning with all of the staff so that everybody's aware of what's going on. That is one thing that we do on a day‐to‐day basis that has come out of Lean’. (#6).

Another participant spoke of a resource person that coached her in bring the project to fruition and collaborated with staff, patients and families in making a meaningful change:

…we just needed some coaching to use some of the Lean methodology to tweak out what we needed. We were not assigned any resources other than that one person, and I thank my lucky stars for that… I was able to pick the brain of my Kaizen promotion office person and use the tools; I would describe what I wanted to do and she'd say, ‘I think this might work.’ (#10).

The majority of participants seemed to go through the motions of engaging with staff on the Gemba walk and being part of huddles, there was little evidence suggesting there was a higher purpose and value to this action, such as developing staff learning and action on the Lean process.

## 4 DISCUSSION

Our findings revealed that in this Western Canadian study, the Lean management system was not effectively implemented into organisational cultures; participants were unable to overcome organisational barriers and work demands to gain an understanding of and create the conditions for sustaining the Lean system.

There was limited focus on supporting managers through investing in education and learning about the Lean philosophy or promoting an organisational culture that supported Lean leadership (Aji & Rapsaniotis, 2017). The approach to Lean in the initial implementation was focused on utilizing the tools, and as such, it was difficult, if not impossible, to create and sustain improvements without a manager's knowledge and commitment ‘to redefine institutionalized ways of working’ (Waring & Bishop, 2010, p. 1334).

Our findings confirm that nurse managers made efforts to align their leadership style with features of Lean by performing the requisite Gemba walks, reputed to motivate and engage staff in problem‐solving practice issues. But, a limited understanding and knowledge of the Lean system combined with their own burgeoning workloads created additional pressures and limited effective implementation. It is interesting that participants did perceive that Lean had value, but the context in which they were tasked with implementing Lean was overshadowed and dominated by more restraining forces (patient complexities, financial constraints) than driving forces (champions). We agree with previous research calling for organisations to invest adequate resources to engage in process improvement activities (Nicosia, Park, Gray, Yakir, & Hung, 2018).

Some researchers have suggested that motivating others, establishing goals, removing barriers and delegating duties are part of Lean leadership (Dannapfel, Poksinka, & Thomas, 2014; Toussaint & Berry, 2013). For the most part, participants in this study understood that their work was shifting to a greater focus on managing people; they attempted to create conditions for a learning‐enriched work environment by being more visible on units and coaching staff to engage in continuous improvement, but the demands of their other responsibilities were not diminished or delegated, creating untenable workloads (Drotz & Poksinska, 2014). Swedish researchers have identified that to successfully translate Lean into health care settings, there are important differences between jobs in manufacturing and roles in health care that must be understood (Drotz & Poksinska, 2014; Nicosia et al., 2018). The intensive nature of improvement process work and the need to respond to complex dynamic patient needs can create significant additional work for nurses and add significant administrative responsibility for managers.

Participants in the current study also revealed they had the sense that administrators would likely replace Lean with another quality management system, an assumption promoting resistance. When Lean becomes mandated as ‘non‐negotiable despite any dissatisfaction that it….may generate for staff, it is possible resistance may become even more fueled’ (D'Andreamatteo, Ianni, Lega, & Sargiacomo, 2015, p. 14). Adding to this hurdle was the justification that Lean would result in massive savings for the health care system; however, this goal may not be tenable for professionals whose autonomy is constrained by perceived regimented processes and who object to less time spent on direct patient care (Nicosia et al., 2018). As such, Mann (2009) argues that a focus on financial restraint may not result in the intended outcomes of patient quality.

We concur with Aji et al. (2015) who stated that developing leadership capacity that influences the organisational culture is necessary for successful implementation to improve patient outcomes. The findings are corroborated by additional reports that a lack of evidence‐based leadership behaviours at all levels of the organisational hierarchy, including front‐line nurse managers, is a key factor in failed Lean implementation initiatives (Dahlgaard, Pettersen, & Dahlgaard‐Park, 2011; Noori, 2015). A qualitative study with nurses concluded institutions that adopt a Lean management system extensively, across all departments, are positively associated with nurses' second‐order problem‐solving skills—the ability to logically resolve problems by identifying and addressing root causes (Gemmel, Van, Beveren, Landry, & Meijboom, 2019). Interestingly, however, Gemmel et al. (2019) also discovered that second‐order problem solving could thrive among hospital departments that had not implemented a Lean system, if management cultivated a workplace that valued logic and a critical approach to problem solving. We agree that it may be important for future research to determine to what extent successful implementation of Lean systems is related to nurses' higher‐order problem‐solving skills, but actively engaging nurses at the front lines where the expertise resides would be an important preliminary step. We would add that additional human and fiscal resources that provide a comprehensive knowledge of Lean for managers and staff would go a long way to provide traction to affect the sustainability of a change.

## 5 CONCLUSION

Our study found that nurse managers without the requisite knowledge of the Lean management system and adequate resources to build and support a continuous improvement process may not realize positive outcomes. Sustained Lean success requires a change in attitude and behaviours among leadership that influences staff on the unit and, ultimately, the organisational culture.

## 6 IMPLICATIONS FOR NURSING MANAGEMENT

The present study has shown that the implementation of Lean in one province in Canada had a largely negative influence on nurse managers' leadership roles. First, organisations wishing to implement Lean should ensure there are adequate resources to finance the initiative. Second, it is critical to ensure the Lean tools are used in tandem with the strategic elements of organisational readiness and leadership (Burgess & Radnor, 2013). Third, leaders implementing Lean should acknowledge that in the long term, some Lean practices may need to evolve from a focus on tools to a more holistic approach that changes the culture and the organisation, and the important role of the leader in this context. In conclusion, managers require organisational support that aligns with their uncompromising commitment to patient care.

## ETHICAL APPROVAL

BEH#16‐103.

## References

[jonm12898-bib-0001] Aji, K. H. , & Rapsaniotis, S. (2017). Leadership requirements for Lean versus servant leadership in health care: A systematic review of the literature. Journal of Healthcare Leadership, 9, 1–14. 29355240 10.2147/JHL.S120166 PMC5774447

[jonm12898-bib-0002] Aji, K. H. , Visse, M. , & Widdershoven, G. A. M. (2015). Lean leadership: An ethnographic study. Leadership in Health Services, 28(2), 119–134. 25921317 10.1108/LHS-03-2014-0015

[jonm12898-bib-0003] Braun, V. , & Clarke, V. (2006). Using thematic analysis in psychology. Qualitative Research in Psychology, 3(2), 77–101. 10.1191/1478088706qp063oa

[jonm12898-bib-0004] Burgess, N. , & Radnor, Z. (2013). Evaluating Lean in healthcare. International Journal of Health Care Quality Assurance, 26(3), 220–235. 10.1108/09526861311311418 23729126

[jonm12898-bib-0005] Dahlgaard, J. J. , Pettersen, J. , & Dahlgaard‐Park, S. M. (2011). Quality and lean health care: A system for assessing and improving the health of healthcare organisations. Total Quality Management & Business Excellence, 22(6), 673–689. 10.1080/14783363.2011.580651

[jonm12898-bib-0006] D'Andreamatteo, A. , Ianni, L. , Lega, F. , & Sargiacomo, M. (2015). Lean in healthcare: A comprehensive review. Health Policy, 119(9), 1197–1209. 10.1016/j.healthpol.2015.02.002 25737260

[jonm12898-bib-0007] Dannapfel, P. , Poksinka, B. , & Thomas, K. (2014). Dissemination strategy for Lean thinking in health care. International Journal of Health Care Quality Assurance, 27(5), 391–404. 10.1108/IJHCQA-01-2013-0001 25087337

[jonm12898-bib-0009] Dickson, E. W. , Singh, S. , Chueng, D. S. , Wyatt, C. C. , & Nugent, A. S. (2008). Application of Lean manufacturing techniques in the emergency department. Journal of Emergency Medicine, 37(2), 177–182. 10.1016/j.jemermed.2007.11.108 18722732

[jonm12898-bib-0010] Drotz, E. , & Poksinska, B. (2014). Lean in healthcare from employees' perspectives. Journal of Health Organisation and Management, 28(2), 177–195. 10.1108/JHOM-03-2013-0066 25065109

[jonm12898-bib-0011] Fine, B. A. , Golden, B. , Hannam, R. , & Morra, D. (2009). Leading Lean: A Canadian healthcare leader's guide. Healthcare Quarterly, 12(3), 32–41. Retrieved from http://www.rotman.utoronto.ca/-/media/Files/Programs-and-Areas/HealthSector/Research_HQ_vol12_no3_Fine.pdf 10.12927/hcq.2013.20877 19553764

[jonm12898-bib-0012] Gemmel, P. , Van Beveren, S. , Landry, S. , & Meijboom, B. (2019). Problem‐solving behaviour of nurses in a lean environment. Journal of Nursing Management, 27(1), 35–41. 30079975 10.1111/jonm.12646

[jonm12898-bib-0013] Goodridge, D. , Westhorp, G. , Rotter, T. , Dobson, R. , & Bath, B. (2015). Lean and leadership practices: Development of an initial realist program theory. BMC Health Services Research, 15(362), 1–15. 10.1186/s12913-015-1030-x 26345184 PMC4562348

[jonm12898-bib-0014] Graban, M. (2009). Lean hospitals: Improving quality, patient safety, and employee satisfaction. New York: NY: Taylor & Francis Group.

[jonm12898-bib-0015] Hamilton, J. , Verrall, T. , Maben, J. , Griffiths, P. , Avis, K. , Baker, G. R. , & Teare, G. (2014). One size does not fit all: A qualitative content analysis of the importance of existing quality improvement capacity in the implementation of Releasing Time to Care: The Productive Ward™ in Saskatchewan, Canada. BMC Health Services Research, 14(1), 642 10.1186/s12913-014-0642-x 25547227 PMC4279911

[jonm12898-bib-0016] Hines, P. , Found, P. , Griffiths, G. , & Harrison, R. (2008). Staying Lean: Thriving not just surviving. Cardiff, UK: Lean Enterprise Research Centre.

[jonm12898-bib-0017] Johnson, J. E. , Smith, A. L. , & Mastro, K. A. (2012). From Toyota to the bedside: Nurses can led the lean way in health care reform. Nursing Administration Quarterly, 36(3), 234–242. 10.1097/NAQ.0b013e318258c3d5 22677964

[jonm12898-bib-0018] Kinsman, L. , Rotter, T. , Stevenson, K. , Bath, B. , Goodridge, S. , Harrison, L. , … Westhorp, G. (2014). “The largest Lean transformation in the world”: The implementation and evaluation of lean in Saskatchewan healthcare. Healthcare Quarterly, 17(2), 29–32. 25191805 10.12927/hcq.2014.23880

[jonm12898-bib-0019] Knowles, S. , & Barnes, I. (2013). Lean laboratories: Laboratory medicine needs to learn from other industries how to deliver more for less. Journal of Clinical Pathology, 66(8), 635–637. 10.1136/jclinpath-2013-201624 23681948

[jonm12898-bib-0020] Lincoln, Y. S. , & Guba, E. G. (1985). Naturalistic inquiry. Beverly Hills, CA: Sage.

[jonm12898-bib-0021] Mann, D. (2009). The missing link: Lean leadership. Frontiers of Health Services Management, 26(1), 15–26. Retrieved from http://dmannlean.com/pdfs/The%20Missing%20Link_Lean%20Leadership_DWMann.pdf 19791484

[jonm12898-bib-0023] Nicosia, F. M. , Park, L. G. , Gray, C. P. , Yakir, M. J. , & Hung, D. Y. (2018). Nurses' perspectives on Lean redesigns to patient flow and inpatient discharge process efficiency. Global Qualitative Nursing Research, 5, 2333393618810658 10.1177/2333393618810658 30480041 PMC6249655

[jonm12898-bib-0024] Noori, B. (2015). Identifying critical issues in lean implementation in hospitals. Hospital Topics, 93(2), 44–52. 10.1080/00185868.2015.1052299 26185933

[jonm12898-bib-0025] Patton, M. Q. (2015). Qualitative research & evaluation methods (4th ed.). Thousand Oaks, CA: Sage.

[jonm12898-bib-0027] Poksinska, B. , Swartling, D. , & Drotz, E. (2013). The daily work of Lean leaders – Lessons from manufacturing and healthcare. Total Quality Management & Business Excellence, 24(7–8), 886–898. 10.1080/14783363.2013.791098

[jonm12898-bib-0029] Rotter, T. , Kinsman, L. , Bath, B. , Goodridge, D. , Harrison, L. , Dobson, R. , … Westhorp, G. (2014). A first phase evaluation of Saskatchewan's Lean transformation. Final report. Saskatoon, Canada: University of Saskatchewan, College of Pharmacy and Nutrition Retrieved from http://research-groups.usask.ca/rotter/documents/select-publications/Lean%20Report%20-%20Full.pdf

[jonm12898-bib-0032] Steed, A. (2012). An exploration of the leadership attributes and methods associated with successful Lean system deployments in acute care hospitals. Quality Management in Health Care, 21(1), 48–58. 22207019 10.1097/QMH.0b013e318241825c

[jonm12898-bib-0033] Titzer, J. L. , & Shirey, M. (2013). Nurse manager succession planning: A concept analysis. Nursing Forum, 48(3), 155–164. 10.1111/nuf.12024 23889194

[jonm12898-bib-0034] Toussaint, J. S. , & Berry, L. L. (2013). The promise of lean in health care. Mayo Clinic Proceedings, 88(1), 74–82. 10.1016/j.mayocp.2012.07.025 23274021

[jonm12898-bib-0035] Waring, J. J. , & Bishop, S. (2010). Lean healthcare: Rhetoric, ritual and resistance. Social Science & Medicine, 71(7), 1332–1340. 20702013 10.1016/j.socscimed.2010.06.028

